# Tetrahexyldecyl Ascorbate (THDC) Degrades Rapidly under Oxidative Stress but Can Be Stabilized by Acetyl Zingerone to Enhance Collagen Production and Antioxidant Effects

**DOI:** 10.3390/ijms22168756

**Published:** 2021-08-15

**Authors:** William R. Swindell, Manpreet Randhawa, Geovani Quijas, Krzysztof Bojanowski, Ratan K. Chaudhuri

**Affiliations:** 1 Department of Internal Medicine, The Jewish Hospital, Cincinnati, OH 45236, USA; 2 Sytheon Ltd., Boonton, NJ 07005, USA; manpreet@sytheonltd.com (M.R.); ratan@sytheonltd.com (R.K.C.); 3 Sunny BioDiscovery Inc., Santa Paula, CA 93060, USA; geovani@sunnybiodiscovery.com (G.Q.); kbojanowski@sunnybiodiscovery.com (K.B.)

**Keywords:** acetyl zingerone, ascorbate, collagen, skin aging, tetrahexyldecyl ascorbate, vitamin C

## Abstract

Tetrahexyldecyl Ascorbate (THDC) is an L-ascorbic acid precursor with improved stability and ability to penetrate the epidermis. The stability and transdermal penetration of THDC, however, may be compromised by the oxidant-rich environment of human skin. In this study, we show that THDC is a poor antioxidant that degrades rapidly when exposed to singlet oxygen. This degradation, however, was prevented by combination with acetyl zingerone (AZ) as a stabilizing antioxidant. As a standalone ingredient, THDC led to unexpected activation of type I interferon signaling, but this pro-inflammatory effect was blunted in the presence of AZ. Moreover, the combination of THDC and AZ increased expression of genes associated with phospholipid homeostasis and keratinocyte differentiation, along with repression of *MMP1* and *MMP7* expression, inhibition of MMP enzyme activity, and increased production of collagen proteins by dermal fibroblasts. Lastly, whereas THDC alone reduced viability of keratinocytes exposed to oxidative stress, this effect was completely abrogated by the addition of AZ to THDC. These results show that AZ is an effective antioxidant stabilizer of THDC and that combination of these products may improve ascorbic acid delivery. This provides a step towards reaching the full potential of ascorbate as an active ingredient in topical preparations.

## 1. Introduction

L-Ascorbic acid (AA) is essential for skin health and nutritional deficiency of this vitamin leads to the well-characterized skin fragility and bruising seen in scurvy (hypovitaminosis C) [1,2]. Within the dermis, AA is required for the synthesis and post-translational modification of collagen [3], with functions that include hydroxylation of proline and lysine, as well as stimulation of collagen gene expression [4,5]. Interestingly, AA is more abundant in the epidermal layer [6], and, in fact, the epidermis expresses a unique AA transporter (SVCT1) not expressed in other organs [7]. Within the epidermis, the role of AA may parallel that of calcium [8], with pro-differentiation effects that improve barrier function through elevation of filaggrin and normalization of the stratum corneum structure [9]. Such effects are accompanied by an increased abundance of stratum corneum barrier lipids, including glucosylceramides, ceramides, and lipid lamellar structures [10]. These observations have prompted the development of topical formulations with the goal of directly delivering AA to skin to achieve cosmetic or therapeutic effects. Topical AA delivery has been reported to improve some aspects of intrinsic skin aging [11,12,13], although stronger evidence supports a role for AA in the antioxidant-mediated prevention of extrinsic aging due to UV radiation exposure [14,15,16]. Further evidence has supported a role for topically-applied AA or AA-derivatives for treatment of wound healing and scar formation [17,18,19], allergic contact dermatitis [20], hyperpigmentation [21,22,23,24,25], atopic dermatitis [26,27], striae distensae [28], cutaneous malignancies [29,30,31], and psoriasis [32].

Longstanding challenges of topical AA formulations have been related to skin-penetration and the stability of AA as an active ingredient [33]. AA is a water-soluble hydrophilic anion with a high polarity [34], which is repelled by the stratum corneum and can only penetrate skin under acidic conditions (pH < 3.5) [35]. Moreover, upon exposure to ambient air and UV light, AA undergoes oxidation to dehydro-L-ascorbic acid in a reaction facilitated by high temperatures and potentially by vehicle solution properties (e.g., high pH, metal ions, presence of dissolved oxygen) [36]. Additionally, AA can react with singlet oxygen to generate more persistent reactive oxygen species, such as H_2_O_2_, such that AA may function as a pro-oxidant in some circumstances [37,38]. To improve the efficacy of topical delivery, ascorbyl phosphate salts have been developed, which feature an added phosphate group to prevent oxidation [19,39]. These products, however, must undergo an in vivo conversion to AA and tend to have poor skin penetration due to their higher charge densities [35]. Another approach has been to develop combination products to improve the stability and/or transdermal penetration of AA or AA-derivatives [40,41]. For example, a formulation that combined AA with ferulic acid was shown to have improved stability [42], and skin permeability was bolstered by combination of AA with pyridoxine (vitamin B6) [43].

Tetrahexyldecyl ascorbate (THDC) is a lipid-soluble AA precursor esterified with branched chain fatty acid (2-hexyldecanoic acid), which is reported to have an improved stability and ability to penetrate the lipophilic stratum corneum [21,44]. It is essentially an AA pro-drug that can penetrate the dermis where it may then undergo intracellular enzymatic conversion to AA. Moreover, the lipid-soluble property of THDC may facilitate its incorporation into cell membranes to confer added protective benefit [44]. When combined with other ingredients, THDC has been shown to decrease signs of photodamage, such as skin wrinkling [44,45] and reduce hyperpigmentation [21,46]. As such, THDC has become the most popular lipophilic ascorbic acid derivative in the skin care market. Despite its widespread use, however, the stability of THDC in the presence of reactive oxygen species has not been fully demonstrated, and stabilizing antioxidants may be needed to prolong the effective in vivo half-life of THDC. In this respect, acetyl zingerone (AZ) is a previously described antioxidant compound able to neutralize non-radicals, such as singlet oxygen and peroxynitrite anion and radicals, such as hydroxyl and peroxyl [47,48,49]. The ability of AZ to physically quench singlet oxygen, in particular, may prolong THDC half-life and impede production of H_2_O_2_ generated from the reaction between THDC-derived AA and singlet oxygen [37]. The combination of THDC with AZ may therefore have synergistic effects above and beyond those of each compound individually, although interactions between these two antioxidant compounds have not been investigated.

The goals of this study were to investigate the effects of AZ on THDC stability and the mechanism of action. We evaluated the effects of THDC, AZ, and THDC + AZ using cell-free in vitro assays and reconstituted human epidermis (RHE) tissues (EpiDermFT™). The effects of both compounds on RHE tissue cultures were evaluated using in situ oligonucleotide microarrays (Affymetrix Clariom S assays). Our results provide new insights into the effects of THDC in a human skin model and show how these effects can be modulated through combination with AZ as a stabilizing antioxidant.

## 2. Results

### 2.1. THDC Is a Poor Antioxidant That Degrades Rapidly in the Presence of Singlet Oxygen

**Figure 1:**
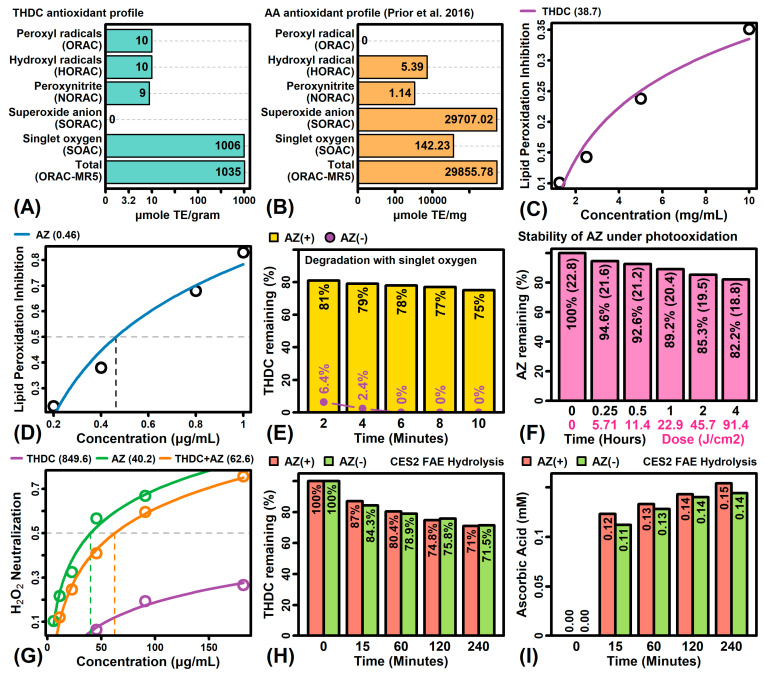
THDC degradation, inhibition of lipid peroxidation, hydrolysis, and antioxidant activity. (**A**) THDC ORAC profile. (**B**) AA ORAC profile. The antioxidant activity against each reactive oxygen species was determined (μmole Trolox equivalents (TE) per gram). The AA ORAC profile shown in (**B**) was reported previously [50]. (**C**,**D**) Inhibition of lipid peroxidation. The degree of inhibition is shown at different THDC or AZ concentrations (top margin: estimated IC50). (**E**) THDC degradation under singlet oxygen. The percentage of remaining THDC is shown under AZ(+) and AZ(−) conditions. (**F**) AZ stability under photooxidation. AZ was exposed to UVA and UVB light for 4 h with the cumulative dose indicated. The percentage of AZ remaining at each time point is shown (absolute quantities in parentheses, μmole TE/gram). (**G**) H_2_O_2_ scavenging activity. The neutralization of H_2_O_2_ was assessed at varying THDC, AZ and THDC + AZ (1:1) concentrations (*n* = 2 replicates). The estimated IC_50_ value is shown (top margin). (**H**,**I**) THDC fatty acid ester hydrolysis with carboxylesterase-2 (CES2). (**H**) The percentage of THDC remaining at each time point and (**I**) the accumulation of ascorbic acid product (mM).

The antioxidant profile of THDC against various reactive oxygen species was evaluated (Figure 1A). This showed the strongest activity against singlet oxygen, with a total oxygen radical absorbance capacity (ORAC) of 1035 μM Trolox equivalents per gram (Figure 1A). This total ORAC value is substantially less than that of AA reported previously (i.e., 29,855.78 μM Trolox equivalents per mg; Figure 1B) [50]. THDC was also able to effectively inhibit lipid peroxidation (IC_50_ 38.7 mg/mL; Figure 1C), although its potency in this regard was much less compared to AZ (IC_50_ = 0.46 µg/mL; Figure 1D). We next used HPLC to monitor THDC degradation under singlet oxygen (Figure 1E). In the absence of AZ, THDC degraded completely by 6 min (Figure 1E). However, in the presence of AZ, there was only a 25% degradation of THDC after 10 min (Figure 1E). Photooxidation analyses showed that AZ was stable under UVA/UVB light exposure, with >80% of AZ remaining following 4 h of exposure (Figure 1F).

Since the reaction between THDC and singlet oxygen generates H_2_O_2_ [37], we next evaluated H_2_O_2_ scavenging capacity of THDC, AZ and THDC + AZ (1:1). This showed that THDC alone has a weak H_2_O_2_ neutralization capacity (IC_50_ 850 µg/mL), whereas AZ or THDC + AZ neutralized H_2_O_2_ more effectively (IC_50_ ≤ 62.6 µg/mL) (Figure 1G). We further evaluated the conversion of THDC to ascorbic acid in the presence of carboxylesterase-2 (CES2), which is the main carboxylesterase expressed by KCs with a role in epidermal prodrug metabolism [51]. From 0 to 240 min, the percentage of THDC remaining was similar in AZ(−) and AZ(+) conditions, with slightly greater accumulation of ascorbic acid under AZ(+) conditions (Figure 1H,I).

### 2.2. THDC Activates Type I Interferon Signaling in the Absence of AZ

**Figure 2:**
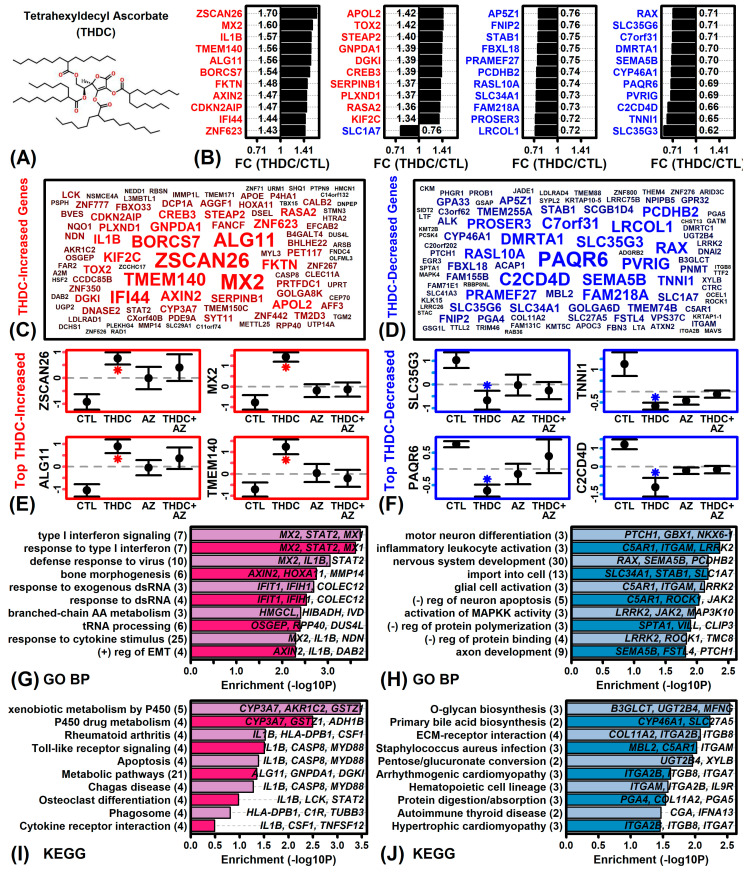
THDC vs. CTL DEG summary. (**A**) THDC structure. (**B**) Top-ranked DEGs. The 44 genes with lowest *p*-value are shown (ranked by FC). (**C**,**D**) DEG clouds. The 100 increased (red) or decreased (blue) genes with lowest *p*-value are shown. Genes with lower *p*-values have larger font. (**E**,**F**) Average expression. Expression of each gene is Z-score normalized (mean = 0; standard deviation = 1). Average expression ±1 standard error is shown for each gene and treatment. An asterisk (*) denotes a significant expression difference (THDC vs. CTL or THDC + AZ vs. AZ; P < 0.05, moderated *t*-statistic). (**G**,**H**) GO BP terms. GO BP terms most strongly enriched among (**G**) increased DEGs and (**H**) decreased DEGs is shown. (**I**,**J**) KEGG terms. KEGG terms most strongly enriched among (**I**) increased and (**J**) decreased DEGs are shown. In (**G**–**J**), the number of genes associated with each term is given in parentheses and exemplar DEGs are listed in the figure.

Microarrays were used to evaluate the effects of THDC on gene expression in the absence of AZ (THDC vs. CTL comparison). Under AZ(−) conditions, THDC altered the expression of 406 DEGs, including 236 THDC-increased DEGs (P < 0.05, FC > 1.25) and 170 THDC-decreased DEGs (P < 0.05, FC < 0.80). Genes most strongly increased by THDC included zinc finger and SCAN domain containing 26 (*ZSCAN26*), MX dynamin like GTPase 2 (*MX2*), ALG11 alpha-1,2-mannosyltransferase (*ALG11*), and transmembrane protein 140 (*TMEM140*) (Figure 2B,C,E). Genes most strongly decreased by THDC included solute carrier family 35 member G3 (*SLC35G3*), troponin I1 slow skeletal type (*TNNI1*), progestin and adipoQ receptor family member 6 (*PAQR6*), and C2 calcium dependent domain containing 4D (*C2CD4D*) (Figure 2B,D,F). As a group, THDC-increased genes were most strongly associated with type I interferon signaling, response to virus, and P450 xenobiotic metabolism (Figure 2G,I). Likewise, THDC-decreased genes were most strongly associated with motor neuron differentiation, inflammatory leukocyte activation, and O-glycan biosynthesis (Figure 2H,J). 

### 2.3. THDC in the Presence of AZ Up-Regulates Phospholipid Homeostasis Genes While Repressing Chemokine Signaling Genes

**Figure 3:**
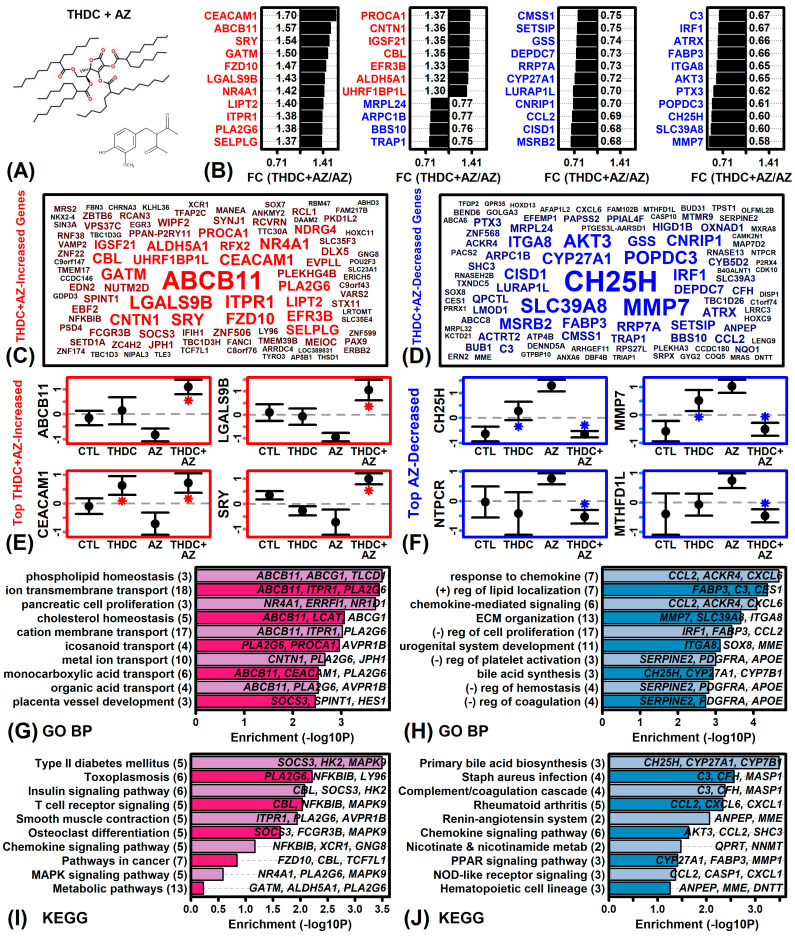
THDC + AZ vs. AZ DEG summary. (**A**) THDC and AZ structures. (**B**) Top-ranked DEGs. The 44 genes with lowest *p*-value are shown (ranked by FC). (**C**,**D**) DEG clouds. The 100 increased (red) or decreased (blue) genes with lowest *p*-value are shown. Genes with lower *p*-values have larger font. (**E**,**F**) Average expression. Expression of each gene is Z-score normalized (mean = 0; standard deviation = 1). Average expression ±1 standard error is shown for each gene and treatment. An asterisk (*) denotes a significant expression difference (THDC vs. CTL or THDC + AZ vs. AZ; P < 0.05, moderated t-statistic). (**G**,**H**) GO BP terms. GO BP terms most strongly enriched among (**G**) increased DEGs and (**H**) decreased DEGs are shown. (**I**,**J**) KEGG terms. KEGG terms most strongly enriched among (**I**) increased and (**J**) decreased DEGs are shown. In (**G**–**J**), the number of genes associated with each term is given in parentheses and exemplar DEGs are listed in the figure.

We next used microarrays to evaluate THDC responses in the presence of AZ (THDC + AZ vs. AZ comparison). Under AZ(+) conditions, THDC altered expression of 347 DEGs, including 168 THDC-increased DEGs (P < 0.05, FC > 1.25) and 179 THDC-decreased DEGs (P < 0.05, FC < 0.80). Genes most strongly increased by THDC included ATP binding cassette subfamily B member 11 (ABCB11), galectin 9B (LGALS9B), CEA cell adhesion molecule 1 (CEACAM1), and sex determining region Y (SRY) (Figure 3B,C,E). Genes most strongly decreased by THDC included cholesterol 25-hydroxylase (CH25H), matrix metallopeptidase 7 (MMP7), nucleoside-triphosphatase cancer-related (NTPCR), and methylenetetrahydrofolate dehydrogenase 1 like (MTHFD1L) (Figure 3B,D,F). As a group, THDC-increased DEGs were associated with phospholipid homeostasis, ion transmembrane transport, and type 2 diabetes mellitus (Figure 3G,I). Likewise, THDC-decreased DEGs were associated with response to chemokine, positive regulation of lipid localization, and chemokine signaling (Figure 3H,J).

### 2.4. MMP7 and NQO1 Have Differential Responses to THDC in AZ(−) and AZ(+) Conditions

**Figure 4:**
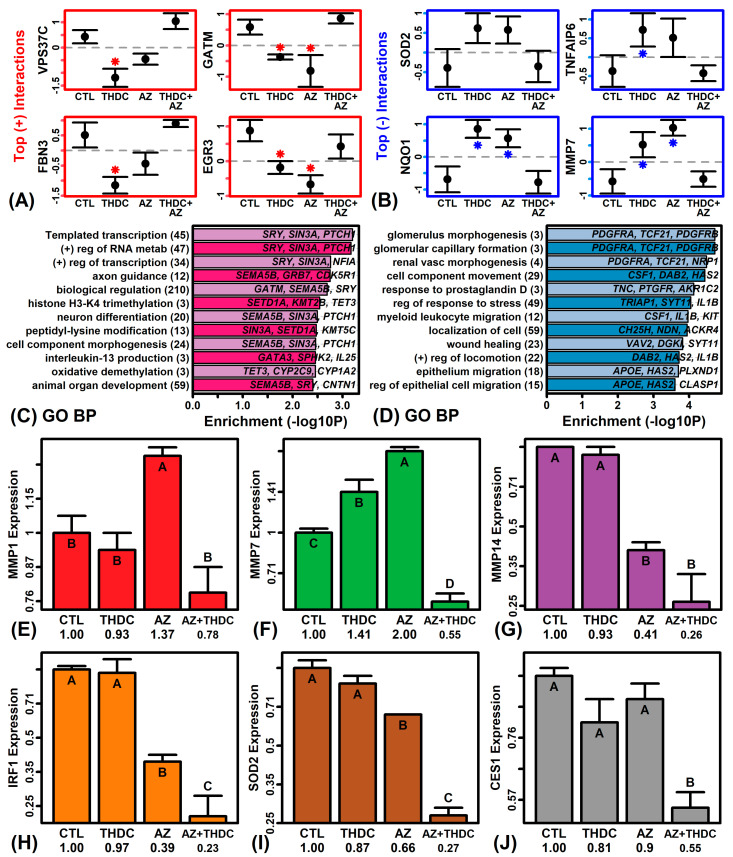
Genes with differential responses to THDC in AZ(−) and AZ(+) conditions. (**A**) Top-ranked genes with a positive interaction effect (i.e., up-regulated by THDC in presence of AZ but not in the absence of AZ; *P < 0.05, comparison to CTL). (**B**) Top-ranked genes with a negative interaction effect (i.e., down-regulated in the presence of AZ but not in the absence of AZ). In (**A**,**B**), average gene expression is shown (±1 standard error of the mean). (**C**) GO BP terms enriched among genes with a positive interaction pattern. (**D**) GO BP terms enriched among genes with a negative interaction pattern. In (**C**,**D**), the number of genes associated with each GO BP term is given in parentheses (left margin) and exemplar genes are listed. (**E**–**J**) Real-time quantitative PCR (RT-PCR) results. Average expression is shown for each group ±1 standard error (*n* = 2 per group). Groups without the same letter differ significantly (P < 0.05, Fisher’s least significant difference). Relative expression was calculated using hypoxanthine phosphoribosyltransferase 1 (*HPRT1*) as a reference and then normalized to the CTL treatment.

Further analyses were performed to identify genes differentially regulated by THDC in AZ(+) and AZ(−) conditions. This identified 302 genes with a positive interaction pattern (P < 0.05) and 392 genes with a negative interaction pattern (P < 0.05). A positive interaction pattern is characterized by genes for which expression is up-regulated more strongly by THDC in AZ(+) compared to AZ(−) conditions (Figure 4A). A negative interaction pattern is characterized by genes for which expression is down-regulated more strongly by THDC in AZ(+) conditions compared to AZ(−) conditions (Figure 4B).

Examples of genes with a positive interaction pattern included VPS37C subunit of ESCRT-I (*VPS37C*), glycine amidinotransferase (*GATM*), fibrillin 3 (*FBN3*), and early growth response 3 (*EGR3*) (Figure 4A). Such genes were most strongly associated with transcription, methylation, IL-13 production, and oxidative demethylation (Figure 4C). Examples of genes with a negative interaction pattern included superoxide dismutase 2 (*SOD2*), TNF alpha induced protein 6 (*TNFAIP6*), NAD(P)H dehydrogenase quinone 1 (*NQO1*), and matrix metallopeptidase 7 (*MMP7*) (Figure 4B). These genes were most strongly associated with response to stress and leukocyte/epithelium migration (Figure 4D). The negative interaction pattern was confirmed for a subset of genes using RT-PCR assays (i.e., *MMP1*, *MMP7*, *IRF1*, *SOD2*, and *CES1*; Figure 4E–J).

### 2.5. AZ Moderates Pro-Inflammatory Gene Expression Changes Observed with THDC Treatment

**Figure 5:**
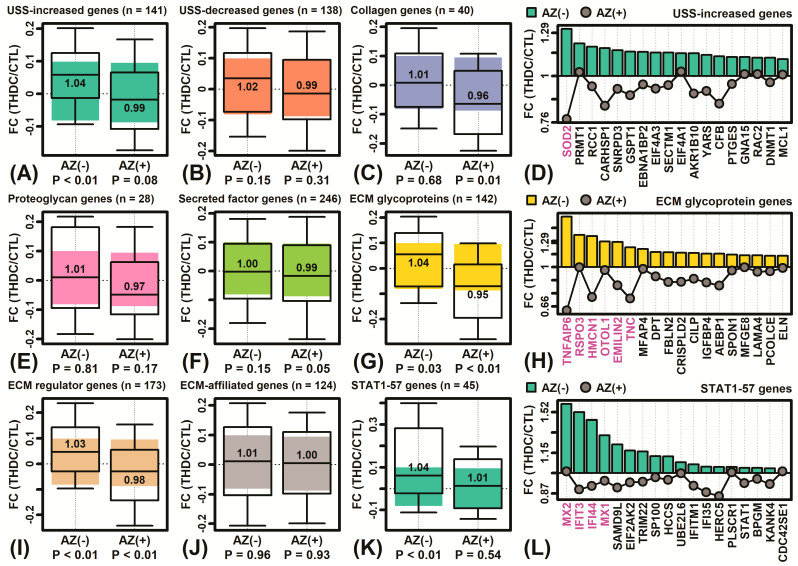
Unhealthy skin signature (USS), matrisome category, and STAT1-57 gene expression responses. (**A**–**C**,**E**–**G**,**I**–**K**) Boxplots show the median and interquartile range of fold-change estimates in AZ(−) and AZ(+) conditions for genes in each category (whiskers: 10th to 90th percentile). The number of genes in each category is indicated (top margin). For each boxplot, the colored region outlines the interquartile range observed for all expressed genes not included within the indicated category (*p*-value: Mann Whitney U test). (**D**,**H**,**L**) Example genes from selected categories with differential responses to THDC in AZ(−) and AZ(+) conditions. Genes are ranked based upon fold-change (THDC/CTL). Genes significantly altered by THDC in AZ(−) or AZ(+) conditions are shown in magenta font (P < 0.05, bottom margin).

The effects of THDC on pre-defined gene sets were evaluated (Figure 5). In AZ(−) conditions, THDC up-regulated genes increased as part of the unhealthy skin signature (P < 0.01), which is a set of genes increased in diverse types of inflammatory skin disease (e.g., *SOD2*, *PRMT1*, *RCC1*; Figure 5A,D) [52]. However, this effect was absent under AZ(+) conditions (Figure 5A). Likewise, under AZ(−) conditions, THDC increased expression of genes belonging to the STAT1-57 module (P < 0.01), which is a set of genes activated by interferon signaling with elevated expression in inflammatory skin disease (e.g., *MX2*, *IFIT3*, *IFI44*; Figure 5K,L) [53]. The effect, however, was not seen under AZ(+) conditions (Figure 5K). Otherwise, THDC decreased expression of genes belonging to some matrisome categories [54] in the AZ(+) condition (e.g., collagen genes, ECM glycoproteins, ECM regulator genes; Figure 5C,G,I) but not in the AZ(−) condition. Examples of ECM glycoprotein genes with increased expression in the AZ(−) condition only included TNF alpha induced protein 6 (*TNFAIP6*), hemicentin 1 (*HMCN1*), and tenascin C (*TNC*) (Figure 5H).

### 2.6. The THDC + AZ Combination Triggers Gene Expression Shifts Similar to Those Seen during KC Differentiation

**Figure 6:**
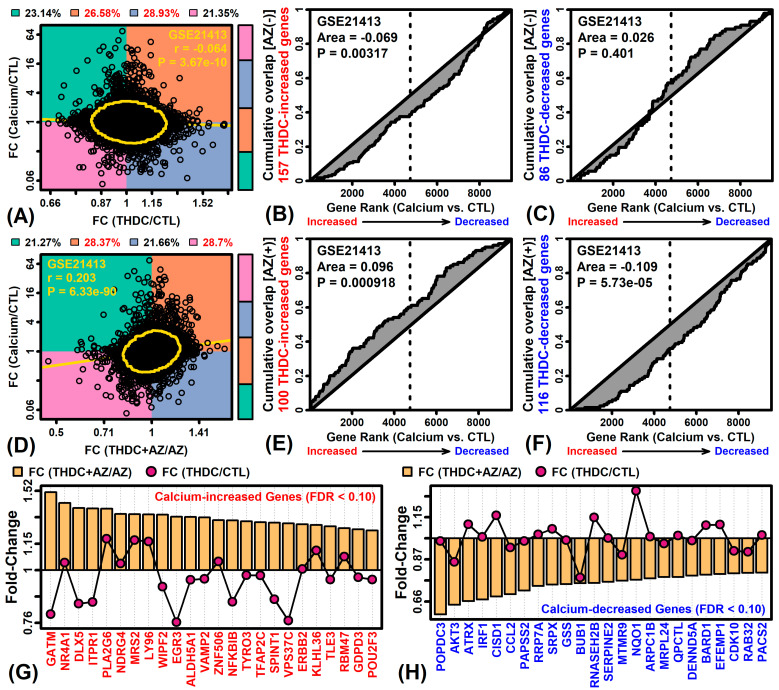
Comparison to gene expression responses in calcium-treated KCs (GSE21413). (**A**,**D**) FC scatterplots. Each point represents an individual gene. The proportion of genes in each quadrant is represented (right sidebar) and shown (top margin; red font: P < 0.05, Fisher’s exact test). The least square regression estimate is shown (yellow line) with Spearman correlation and corresponding *p*-value (yellow ellipse: middle 90% of genes, Mahalanobis distance). (**B**,**C**,**E**,**F**) GSEA analyses. Figures show the cumulative overlap between THDC-regulated genes (±AZ) (P < 0.05) and a list of genes ranked based upon response to calcium (GSE21413). The area between the curve and diagonal is shown with corresponding *p*-value (Mann–Whitney U test). (**G**) Calcium-increased genes (FDR < 0.10) decreased by THDC + AZ. (**H**) Radiation-decreased genes (FDR < 0.10) increased by THDC + AZ. In (**G**,**H**), FC estimates are shown for each gene (THDC/CTL) with and without AZ.

We next evaluated the effects of THDC on the expression of genes regulated during KC differentiation. Genes with expression altered during KC differentiation were identified from a prior microarray study that compared differentiating KCs (high calcium medium, 7 days) to proliferating KCs (sub-confluent cells) (GSE21413). In the absence of AZ, effects of THDC on gene expression were negatively correlated with those observed during KC differentiation (*r* = −0.064, P = 3.67 × 10^−10^; Figure 6A). Consistent with this, the set of THDC-increased genes evaluated in both studies tended to be decreased by high-calcium medium (Figure 6B), whereas no significant trend was observed for THDC-decreased genes (Figure 6C). These effects of THDC, however, differed in the presence of AZ. Under the AZ(+) condition, effects of THDC on gene expression were positively correlated with those seen during KC differentiation (*r* = 0.203, P = 6.33 × 10^−90^; Figure 6D). Consistent with this, THDC-increased genes [AZ(+) condition] tended to be up-regulated in high-calcium medium (P < 0.01; Figure 6E), and THDC-decreased genes (AZ(+) condition) tended to be down-regulated in high-calcium medium (P < 0.01; Figure 6F). Examples of calcium-increased genes up-regulated by THDC + AZ included glycine amidinotransferase (*GATM*), nuclear receptor subfamily 4 group A member 1 (*NR4A1*), distal-less homeobox 5 (*DLX5*) (Figure 6G), and examples of calcium-decreased genes down-regulated by THDC + AZ included Popeye domain containing 3 (*POPDC3*), AKT serine/threonine kinase 3 (*AKT3*), and ATRX chromatin remodeler (*ATRX*) (Figure 6H).

### 2.7. The Addition of AZ to THDC Augments Collagen Protein and Inhibits MMP Expression and Activity

**Figure 7:**
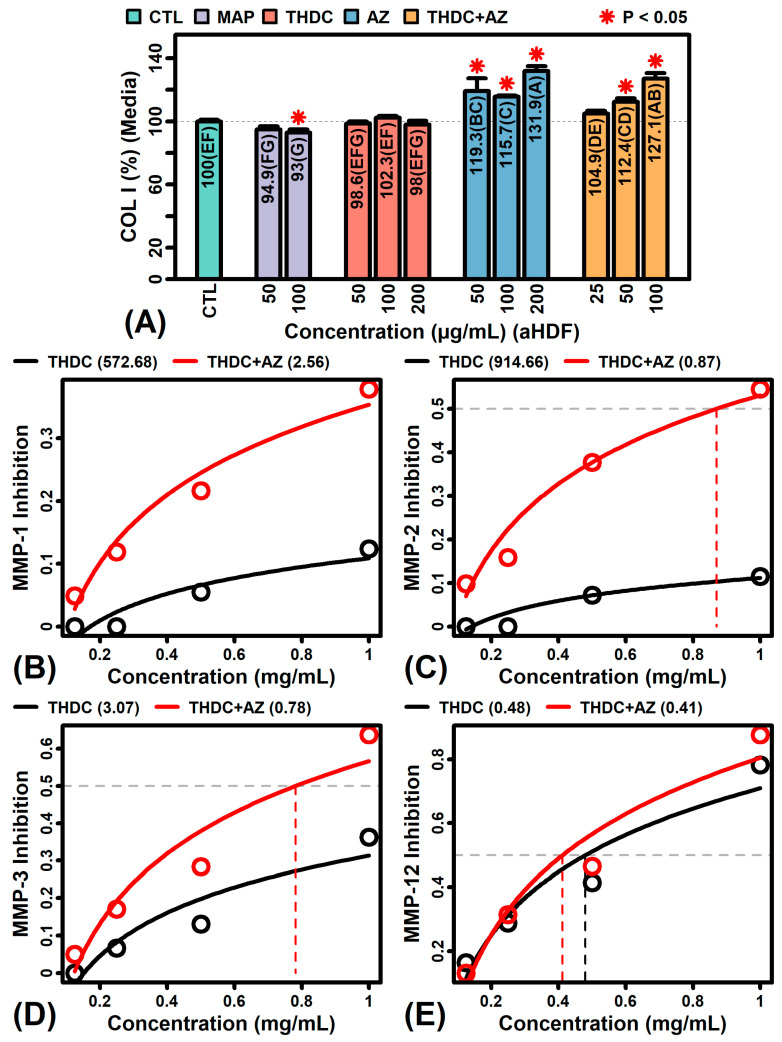
Effects of THDC (±AZ) on collagen I protein and matrix metalloproteinase (MMP) enzymes. (**A**) Collagen I protein (COL I). Collagen I abundance was measured using sandwich ELISA assays with magnesium ascorbyl phosphate (MAP) as a positive control (*n* = 6–16 per group). Colorimetric signals were first normalized to cell numbers (sulforhodamine B assay) and then to the non-treated CTL group. Treatments not sharing the same letter differ significantly (P < 0.05, Fisher’s least significant difference; * P < 0.05, compared to CTL group). (**B**–**E**) MMP inhibition. Inhibition is shown at various THDC concentrations. Experiments were performed with (red) and without (black) AZ (top margin: estimated IC_50_). Results were averaged over two replicate studies.

We next evaluated effects of THDC, AZ and THDC + AZ on production of COL I protein in human dermal fibroblasts (Figure 7A). AZ elicited a significant 15–31% extracellular increase in COL I production at all concentrations tested in adult fibroblasts, whereas the combination THDC + AZ increased COL I protein by 12–27% at the highest concentrations tested (50 and 100 µg/mL; Figure 7A). In contrast, THDC alone not significantly alter COL I protein levels (Figure 7A). THDC also did not increase extracellular COL I protein in neonatal fibroblasts, although in neonatal cells there was only a modest 7% increase in COL I seen with AZ treatment (50 µg/mL; P < 0.05), without significant effect of THDC + AZ treatment (25–100 µg/mL; data not shown).

THDC alone did yield a small (<10%) but significant increase in cellular (but not extracellular) levels of COL IV and COL VI proteins in neonatal and adult fibroblasts (Figure S1). However, AZ significantly increased both cellular and extracellular COL IV and VI proteins in adult and neonatal fibroblasts (Figure S1), with a substantial (>50%) increase observed in extracellular COL IV from neonatal cells (Figure S1B). The effects of THDC + AZ were intermediate to those of THDC and AZ alone, but in some cases THDC + AZ increased extracellular COL IV or VI abundance when THDC alone had no effect (e.g., see Figure S1B,H). We next used cell-free assays to evaluate the relative effects of THDC and THDC + AZ on matrix metallopeptidase (MMP) enzyme activity. This showed that the combination THDC + AZ led to more potent inhibition of MMP-1, MMP-2, and MMP-3 activity as compared to AZ alone (Figure 7B–E).

### 2.8. The Combination AZ + THDC Improves Survival of KCs Treated with Oxidative Stress

**Figure 8:**
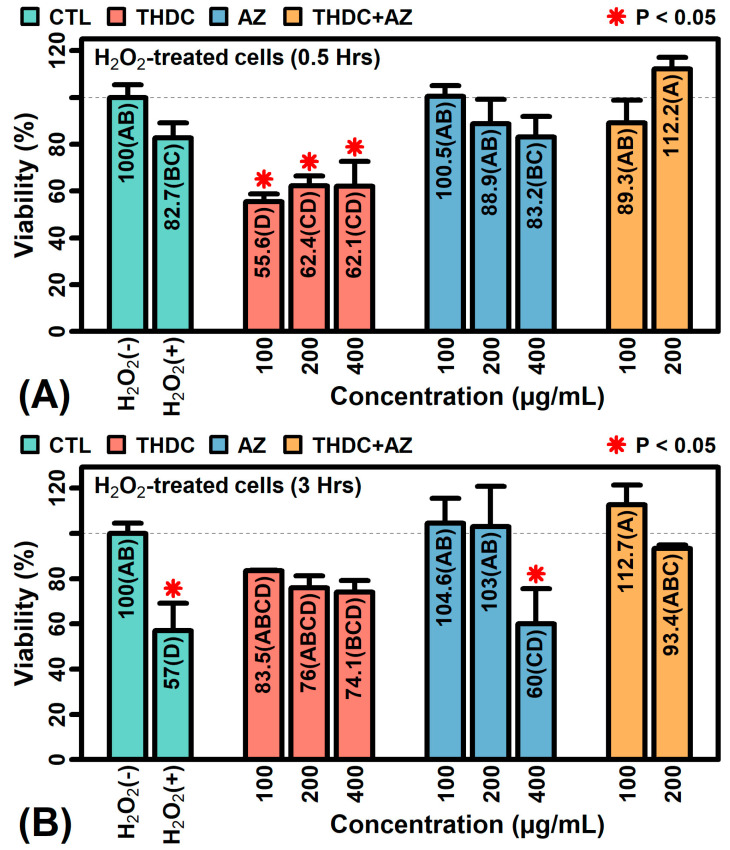
Effects of THDC (±AZ) on viability of KCs exposed to oxidative stress. (**A**) The 0.5-h H_2_O_2_ exposure (*n* = 3–7 per group). (**B**) The 3-h H_2_O_2_ exposure (*n* = 2–4 per group). Cells were incubated with H_2_O_2_ (10 mM) for 0.5 or 3 h and viability was assessed. Colorimetric signals are normalized to the non-treated CTL. Groups not sharing the same letter differ significantly (P < 0.05, Fisher’s least significant difference; * P < 0.05, comparison to non-treated CTL group).

We treated HaCaT KCs with hydrogen peroxide (H_2_O_2_) for 0.5 or 3 h and evaluated the effects on KC viability (Figure 8). Both treatment periods reduced KC viability although the reduction was only significant after 3 h (P < 0.05, Figure 8B). After 0.5 h of H_2_O_2_ treatment, the addition of THDC reduced viability further, whereas AZ restored viability, and the highest overall survival was seen in KCs treated with the THDC + AZ combination (at doses of 200 µg/mL each; Figure 8A). After 3 h of H_2_O_2_ treatment, the addition of THDC did promote a non-significant increase in viability, although a stronger a significant increase was seen with AZ, and again the highest overall survival was observed in cells treated with the THDC + AZ combination (at doses of 100 µg/mL each; Figure 8B).

## 3. Discussion

The topical delivery of L-Ascorbic acid (AA) has been a challenge due to its poor stability and dermal penetration. Formulations that include the AA pre-cursor tetrahexyldecyl ascorbate (THDC) have gained widespread use [21,44,45,46], but THDC may itself lack stability within the dermal microenvironment. This study showed that THDC has limited oxygen radical absorbance capacity and undergoes rapid degradation when exposed to singlet oxygen (Figure 1D). This degradation, however, could be prevented by the addition of acetyl zingerone (AZ) as a stabilizing antioxidant (Figure 1D). Moreover, whereas treatment of RHE cultures with THDC alone stimulated expression of type I interferon genes [55], the unhealthy skin gene signature [52], and the pro-inflammatory STAT1-57 gene module [53], all these effects were abrogated in the presence of AZ. Finally, although THDC did not prevent loss of viability in HaCaT KCs exposed to oxidative stress, the combination THDC + AZ was fully protective (Figure 8). These results support a synergistic mechanism by which AZ can stabilize THDC to facilitate delivery of AA into the dermis. This suggests a novel strategy that can be used to maximize the potential of AA as a topical ingredient in skin care formulations.

The current study showed that AZ was more effective than THDC at directly neutralizing hydrogen peroxide (Figure 1F). This most likely explains why THDC alone did not rescue the viability of H_2_O_2_-treated KCs, whereas the combination of THDC + AZ restored KC viability (Figure 8). Under normal conditions, excess formation of H_2_O_2_ within cells is removed by catalase, which converts H_2_O_2_ into water and molecular oxygen [56]. However, catalase becomes depleted due to sun exposure, with seasonal variation corresponding to low activity in summer and higher activity in winter [57,58]. Recovery of catalase following UVA exposure is also diminished in the skin of older subjects, which contributes to extrinsic skin aging [59]. To compensate for this decline in antioxidant defense, the use of topical AA or AA precursors has been proposed [60], but THDC alone may be counterproductive since even more H_2_O_2_ may be generated as a degradation product [37]. Our results suggest that the use of combined THDC-AZ formulations may provide a more stable formulation to maximize ascorbate release. Moreover, our results show that AZ maintained its antioxidant capacity over 4 h of continual exposure to simulated day light (Figure 1E), which is tantamount to a full day of intermittent sun exposure under non-extreme conditions [61]. This long-lasting antioxidant function may be needed to ensure adequate AA release and free radical scavenging activity for THDC + AZ combination products applied to sun-exposed areas of skin (e.g., arms, hands and face).

The effects of THDC on gene expression have not been evaluated previously, but our study has demonstrated differences in THDC bioactivity depending upon whether AZ is present or absent as a co-ingredient. For example, treatment of RHE with the combination THDC + AZ elicited gene expression responses similar to those observed in calcium-stimulated KCs (Figure 6D–E) and further increased expression of genes associated with phospholipid homeostasis (Figure 3G). On the other hand, treatment of RHE with THDC alone tended to elicit gene expression changes opposite to those seen in calcium-treated KCs (Figure 6A–C). Pro-differentiation effects of AA in KCs have been described previously [10,62]. It has been noted that effects of AA mimic those of calcium [8] and the addition of AA to cell culture medium does indeed improve lipogenesis to support maintenance of the stratum corneum barrier [10]. The differential effects of THDC we observed may therefore be explained by stabilization of THDC in AZ(+) conditions, leading to enhanced release of THDC-derived AA into the epidermal and dermal microenvironments, thereby favoring a pro-differentiation response similar to that seen with calcium treatment of KCs [63]. This suggests that the THDC + AZ combination may have practical benefits promoting epidermal barrier recovery, which may be useful for applications such as xerosis and wound healing [64].

A surprising result from this study was that treatment of RHE tissue with THDC alone led to up-regulation of type I interferon genes such as *MX1*, *MX2* and *STAT2* (Figure 2G). This was not seen, however, when THDC was combined with AZ (Figure 3G). The type I interferon pathway plays an important role in antiviral defense responses and appears to be non-specifically activated in a broad spectrum of skin diseases [65]. Consistent with this, treatment of RHE with THDC alone (but not THDC + AZ) led to up-regulation of genes belonging to the “unhealthy skin signature” [52] (Figure 5A) as well as the STAT1-57 gene module (Figure 5K), which is also activated under pathological conditions such as wounding and skin cancers [53]. To our knowledge, no prior study has demonstrated a detrimental pro-inflammatory effect of topically-applied THDC as a standalone ingredient [21,44,45,46]. It is possible that degradation of THDC leads to increased H_2_O_2_ production, which contributes to an overall imbalance between antioxidant and prooxidant factors that results in downstream activation of the type I interferon pathway [66]. This may have reciprocal interactions with the process of KC differentiation [67], with type I interferon pathway activation suppressing differentiation [68], and differentiation in turn suppressing type I interferon pathway activation [69,70]. Through these mechanisms, the combination of AZ with THDC may buffer against aberrant activation of the type I interferon pathway that would otherwise be triggered by THDC alone.

The link between AA and tertiary collagen structure has been known for decades [3] and it is now recognized that AA can also stimulate collagen mRNA synthesis [4,5]. In this study, THDC alone had no significant effect on synthesis of extracellular COL I protein in adult fibroblasts, whereas AZ and the combination THDC + AZ significantly increased COL I protein levels (Figure 7A). Likewise, cellular and extracellular COL IV and VI levels were generally higher in AZ-treated compared to THDC-treated fibroblasts, with THDC + AZ treatment leading to an intermediate increase in COL IV and VI proteins (Figure S1). THDC also had inhibitory activity against MMP-1, MMP-2, MMP-3 and MMP-12 that was bolstered by AZ (Figure 7B–E). Stabilization of THDC by AZ may favor improved collagen synthesis through increased bioavailability of AA. This would increase collagen mRNA synthesis [4,5,71,72] and prevent accumulation of underhydroxylated procollagen protein to bolster procollagen mRNA translation [72]. Likewise, AA derivatives inhibit matrix metalloproteinase-1 (MMP-1) activity in normal human fibroblasts [73], and in one study AA decreased MMP-1 and MMP-2 protein abundance by 20% in UVA-treated human dermal fibroblasts [74]. A nutrient mixture containing AA also inhibited secretion of MMP-9 monomer and dimer in a broad set of cancer cell lines [75], and similar MMP inhibitory activity has been observed in other human and animal models [76,77]. The effects of AZ-stabilized THDC observed in this study thus appear consistent with prior work demonstrating links between AA release, collagen synthesis, and MMP inhibition [4,5,71,72,73,74,75,76,77,78].

THDC has become one of the most widely used L-ascorbic acid precursors, but evidence demonstrating its stability under realistic in vivo conditions is lacking. This study shows that THDC alone is a weak antioxidant with limited capacity for sustained AA release when applied topically. However, through combination with AZ as a stabilizing antioxidant, THDC degradation is slowed, leading to improved antioxidant activity, activation of KC differentiation pathways, and endogenous collagen production. The observed synergy between THDC and AZ as co-ingredients supports their combination in topical formulations.

## 4. Materials and Methods

### 4.1. THDC Antioxidant Capacity

The oxygen radical absorbance capacity (ORAC) of THDC was evaluated using previously described methods [50,79]. ORAC measures the capacity of antioxidants to protect fluorescent protein from damage by free radicals. In this assay, 2,2′-Azobis (2-amidino-propane) dihydrochloride (AAPH) (Sigma, St. Louis, MO, USA) was used as the source of peroxyl radical, which is generated from the spontaneous decomposition of AAPH at 37 °C. Fluorescein (Sigma, St. Louis, MO, USA) was chosen as the target protein, since loss of fluorescence indicates the extent of damage from its reaction with peroxyl radical. The protective effect of the antioxidants was measured by assessing the fluorescence time/intensity area under the curve (AUC) of the sample compared to a blank without antioxidant compounds. Trolox (Sigma, St. Louis, MO, USA) was used as the calibration standard.

The HORAC (Hydroxyl radical scavenging capacity) assay used cobalt fluoride (Sigma, St. Louis, MO, USA) and picolinic acid (Sigma, St. Louis, MO, USA) as reactants with hydrogen peroxide. This reaction causes fluorescent probe decay that is inhibited by antioxidants, with the degree of inhibition quantified based upon the AUC method.

The NORAC (Peroxynitrite scavenging capacity) procedure measures the peroxynitrite radical. In this assay, 3-morpholinosyndnonimine hydrochloride (SIN-1) (Cayman Chemical, Ann Arbor, MI, USA) was used as the source for the peroxynitrite radical, which is generated from the spontaneous decomposition of SIN-1 at 37 °C. Dihydrorhodamine-123 (Sigma, St. Louis, MO, USA) was the chosen target protein.

The superoxide anion scavenging assay (S-ORAC) used hydroethidine (HE) (Polysciences, Inc., Warrington, PA, USA) to measure the O2- scavenging capacity. O2- radicals were generated by the mixture of xanthine (Sigma, St. Louis, MO, USA) and xanthine oxidase (Sigma, St. Louis, MO, USA). Nonfluorescent HE was oxidized by O2- to form a species of unknown structure that emits fluorescence signal at 586 nm.

The singlet oxygen scavenging capacity (SOAC) assay generated singlet oxygen in ethanol by the molybdate-catalyzed isproportionation of hydrogen peroxide at 37 °C. Hydroethidine (HE) was oxidized by singlet oxygen to form oxyethidium, which exhibits a strong fluorescence signal at 590 nm. The inhibition of fluorescence increase in the presence of antioxidant was used as an index of antioxidant capacity.

Total ORACMR5 (Sum of peroxyl (ORAC), hydroxyl (HORAC), superoxide anion (SORAC), peroxynitrite (NORAC), and singlet oxygen (SOAC) radicals/oxidants) was expressed as μmoles TE per gram sample (μM TE/g).

### 4.2. Effects of THDC and AZ on Lipid Peroxidation

Lipid peroxidation was evaluated by monitoring the formation of the reactive aldehyde malonaldehyde (MDA) [80]. The effects of THDC on lipid peroxidation were evaluated by first adding 100 μL of 100 mM AAPH (2,2′-azobis(2-amidinopropane) dihydrochloride) into mixtures of squalene and sampled at different concentrations. Mixtures were then incubated at 37 °C overnight. AAPH is an azo compound that undergoes thermo decomposition to generate peroxyl radicals, which then oxidizes fatty acid substrates to generate lipid peroxides. Decomposition of these unstable peroxides results in the formation of malondialdehyde (MDA), which was quantified colorimetrically at 532 nm MDA is a well-established index of lipid peroxidation on fatty acids [81]. The effects of AZ on lipid peroxidation were evaluated using similar methods, except the first step was performed using AMVN (2,2′-azobis (2,4-dimethylvaleronitrile)) rather than AAPH. The IC_50_ estimates were calculated using robust regression as described previously [82].

### 4.3. THDC Degradation under Singlet Oxygen

The degradation of THDC under singlet oxygen was assessed using previously described methods [48]. Experiments were performed using 5 mL of 1 mg/mL THDC mixed with 5 mL 0.16 mg/mL lithium molybdate, 4 mL 0.01 M NaOH, and 5 mL of 0.015% H_2_O_2_. The final pH of the mixed solution was approximately 5.0. A 500 μL sample was taken from the mixture every 2 min and added to 1 mL of curcumin (1 mg/mL) to terminate the reaction. The sample was then analyzed by high-performance liquid chromatography (HPLC) to monitor degradation. To evaluate the effect of AZ, the experiment was repeated in identical fashion, expect the first step was performed using a 1:1 mixture of 5 mL THDC (1 mg/mL) and AZ (1 mg/mL).

### 4.4. Stability of AZ under Photooxidation

The AZ test sample was prepared in 50% ethanol within a quartz cuvette (Hellma Analytics). The cuvette was then placed in a Rayonet RPR-100 photochemical reactor equipped with four RMR 3500 (UVA) and four RMR-3000 (UVB) lamps (Southern New England Ultraviolet Company, Branford, CT, USA) to simulate daylight. A total of 100 μg/mL of sample was irradiated at 31 °C at a dose of 6.35 mW/cm^2^.

### 4.5. H_2_O_2_ Scavenging Activity

H_2_O_2_ scavenging assays were performed as described previously [83,84]. Test samples were dissolved in ethanol and subsequently diluted with PBS (pH 7.40) to reach the desired concentration. The reaction was then initiated by adding 4 mM of H_2_O_2_ to the sample. The reaction was allowed to proceed for 10 min and absorbance was then recorded at 230 nm. The IC_50_ estimates were calculated using robust regression as described previously [82].

### 4.6. THDC Fatty Acid Ester Hydrolysis

The fatty acid ester hydrolysis of THDC with carboxylesterase-2 (CES2) was evaluated using previously described methods [85]. A total of 1 mM THDC was dissolved in a mixture of DMSO (2%) and ethylene glycol (10%) in 20 mM HEPES (pH 7.4). A 20 μL enzyme solution (1 mg/mL) was then added and incubated at 37 °C for 15, 60, 120, and 240 min. The reaction was stopped with methanol and THDC and AA concentrations were measured using a charged aerosol detector. The HPLC mobile phase consisted of 0.1% formic acid in water, 0.1% TFA in methanol, and isopropanol. A Phenomenex (Torrance, CA, USA) Kinetex C18 column was used (4.6 mm × 100 mm, 2.6 μ). To evaluate the effect of AZ on the reaction, the experiment was repeated with 1 mM THDC and AZ dissolved in the DMSO (2%)/ethylene glycol (10%)/20 mM HEPES solution (pH 7.4).

### 4.7. THDC HPLC Analysis

The analysis was performed using an HPLC system with binary gradient UV detector. Reagents included methanol (HPLC grade or equivalent), trifluoroacetic acid (HPLC grade or equivalent), isopropyl alcohol (HPLC grade or equivalent) and orthophosphoric acid (AR grade or equivalent) with THDC standard. An X-Bridge C18 column was used (50 mm × 4.6 mm × 3.5 µm) with 10 µL injection volume and a column/sample temperature of 25 °C. The total runtime was 60 min with a THDC retention time of 12 min. Mobile phase A was generated by mixing trifluoroaetic acid (1.0 mL) and methanol (1 L) and filtering through a 0.45 µm filter with degasification. Isopropyl alcohol was used as mobile phase B. The diluent was a mixture of orthophosphoric acid, methanol and isopropyl alcohol in a ratio of 1.0:500:500 (*v*/*v*/*v*). The system suitability solution was generated by adding 100 mg THDC to a 50 mL volumetric flask with 30 mL of diluent and sonication. Likewise, sample solution was obtained by adding 100 mg of sample to a 50 mL volumetric flask with 30 mL of diluent and sonication. After equilibrating the column, equal volumes of diluent as blank, system suitability solution and sample solutions were injected into the liquid chromatographic system. Chromatograms were recorded and the blank chromatogram was examined for any extraneous peaks. Peaks less than 0.02% in sample and standard chromatograms were disregarded. The purity of sample solution was determined by the area normalization method.

### 4.8. Microarray Profiling of THDC, AZ, and THDC + AZ Expression Responses

Gene expression profiling was performed using EpiDermFT™ tissues (MatTek, Ashland, MA, USA; cat no. EFT-400, lot no. 29376). The experiment included 4 treatments with 5 replicates each (CTL = control, THDC = tetrahexyldecyl ascorbate, AZ = acetyl zingerone; THDC + AZ = tetrahexyldecyl ascorbate with acetyl zingerone in a 1:1 ratio). Test materials were dissolved in DMSO yielding a 20 mg/mL stock solution. Subsequent dilutions of stock solution were performed in distilled water. Samples of test material were added at a concentration of 10 µg/cm^2^ to the top surface of EpiDermFT tissues. After 24 h, tissues were rinsed and RNA was extracted and purified using the RNeasy Plus Mini kit (Qiagen, Germantown, MD, USA; cat. no. 74134) with QiaCube Connect robotic station. Purified total RNA was assessed at 260 and 280 nm using the NanoDrop Lite (Thermo Fisher Scientific, Waltham, MA, USA). The Affymetrix Clariom S array platform was used to quantify genome-wide expression following standard protocols.

Microarray pseudoimages were inspected for evidence of spatial artifact (Figure S2) [86]. No major spatial artifacts were identified besides the expected area of increased intensity arising from Affymetrix internal control probes (Figure S2). Among the 20 samples, 260/280 absorbance ratios were consistent with high purity RNA and ranged from 1.88 to 2.07 (Figure S3A). Probe-level model residuals [87] were centered at zero for each array, with sample THDC-5 notable for higher residual variation (Figure S3B). Normalized unscaled standard errors (NUSE) and relative log expression (RLE) metrics were calculated for each array (Figure S3C–F) [87]. We noted that two samples (THDC-5, AZ-2) had higher NUSE median and IQR values (Figure S3C,D), whereas two other samples (THDC-1, AZ-2) had elevated RLE median and RLE IQR (Figure S3E,F).

The 20 raw microarray data files (CEL files) were normalized using Robust Multichip Average (R package: Oligo) [88]. A batch correction was applied using the ComBat algorithm [89]. This correction was made based upon a division of samples into two batches (*n* = 8 and *n* = 12, respectively) during the RNA extraction step. These normalization and batch correction steps yielded expression intensities for 27,189 probes. Of these, 21,448 probes were annotated with a human gene symbol, which together were associated with 19,525 unique genes. A subset of 20,289 annotated probes associated with 18,411 unique gene names were linked to protein-coding genes (i.e., those genes having an “NP_” or “NM_” prefix in their Refseq identifier). To limit redundancy in the analysis, when multiple probes were associated with the same human gene, the probe with highest average expression among the 20 samples was selected to include in the analysis. Applying this filter yielded 18,411 probes having a one-to-one relationship with 18,411 unique protein-coding genes. For each sample, the 20% of genes with lowest overall expression were considered to have absent expression (3682 genes per sample).

The outForest algorithm [90] was applied to stratify samples in terms of the number of outlier genes identified (Figure S3G). Fewer than 1% of genes were associated with outlying values in each sample, with a maximal outlier percentage of 0.88% for CTL-4 (Figure S3G). A cluster analysis was performed and suggested that THDC-5 may be regarded as an outlying data sample (Figure S3H). THDC-5 also appeared to be an outlier when the 20 samples were plotted with respect to the first 2 principal component axes (Figure S3I). Together, these analyses suggest that sample THDC-5 could be excluded from analyses, based upon elevated NUSE median and IQR (Figure S3C,D) and the outlying pattern seen in cluster and principal component analyses (Figure S3H,I). Subsequent differential expression analyses were therefore performed excluding THDC-5 (*n* = 19 samples total).

### 4.9. Differential Expression Analyses

Differential expression analyses focused on two independent treatment comparisons to evaluate the effects of THDC in AZ(−) conditions (i.e., THDC vs. CTL) and the effects of THDC in AZ(+) conditions (i.e., THDC + AZ vs. AZ). The THDC vs. CTL two-group comparison was performed for 15,044 protein-coding genes with detectable expression in at least 3 of 9 samples. Likewise, the THDC + AZ vs. AZ two-group comparison was performed for 15,105 protein-coding genes with detectable expression in at least 3 of 10 samples. Differential expression tests were performed using moderated t-statistics with empirical Bayes moderation of gene-specific standard errors (R package: limma; function: eBayes).

A second set of analyses was performed in which the data were treated as a 2 × 2 factorial design, with presence/absence of THDC as one factor (1 = THDC present; 0 = THDC absent) and presence/absence of AZ as a second factor (1 = AZ present; 0 = AZ absent). These analyses were carried out for 15,130 genes with detectable expression in at least 5 of 19 samples. This allowed us to evaluate genes for which effects of THDC differ in AZ(−) conditions as compared to AZ(+) conditions (i.e., a significant THDC×AZ interaction effect). Statistical tests were performed using moderated t-statistics (R package: limma; function: eBayes) with a nested interaction formula and estimation of the interaction term using a contrast statement (R package: limma; function: contrasts fit).

For both sets of analyses described above (differential expression and interaction tests), raw *p*-values were corrected for multiple hypothesis testing using the Benjamini-Hochberg method. Raw *p*-value distributions obtained from differential expression tests were approximately uniform with no discernible bias among genes having low or high expression (Figure S4).

### 4.10. Real-Time Quantitative PCR

cDNA was prepared using the AzuraQuant cDNA kit (Azura Genomics, Raynham, MA) (CTL, THDC, AZ, THDC + AZ, *n* = 2 samples per group). The expression of 9 target genes was evaluated using real-time quantitative PCR and the BioRad iCycler iQ detection system. The chosen target genes were *MMP1*, *MMP2*, *MMP7*, *MMP14*, *IRF1*, *IL11B*, *SOD2*, *NQO1*, and *CES1*. PCR primers were purchased from Realtimeprimers (Elkins Park, PA, USA) and reactions were performed using AzuraQuant Green Fast qPCR Mix Fluor (Azura Genomics, Raynham, MA, USA). The ΔΔCt method was used to estimate relative expression with hypoxanthine phosphoribosyltransferase 1 (*HPRT1*) as a reference gene [91].

### 4.11. Effects of THDC (±AZ) on Collagen Production

The effect of THDC (±AZ) on collagen production was evaluated in exponentially growing neonatal and adult human adult dermal fibroblasts (nHDF and aHDF). Test materials were dissolved in DMSO at 20 mg/mL. All further dilutions of stock solution were made in sterile distilled water. Samples were added to nHDF (Cell Applications, San Diego, CA, USA, cat. no. 106K-05n) or aHDF (Cell Applications, San Diego, CA, USA, cat. no. 106K-05a). Cells were cultured in DMEM and 10% FBS. At the end of the experiment, biomarkers were quantified in cells and/or cell culture media. Collagen I quantification was performed using reagents from Southern Biotechnology (Birmingham, AL, USA) following a previously described sandwich ELISA protocol [92,93]. Collagens IV and VI were quantified in formalin-fixed cultures by direct ELISA assays using Southern Biotechnology (Birmingham, AL, USA) biotinylated anti-type IV collagen antibody/streptavidin-HRP (cat. no. 1340-08) and Santa Cruz Biotechnology (Dallas, TX, USA) HRP-conjugated anti-type VI collagen monoclonal antibody (cat. no. Sc-377143HRP). Collagen IV was quantified in cell culture conditioned medium by sandwich ELISA assay using an R&D systems (Minneapolis, MN, USA) anti-collagen IV α1 antibody for capture (cat. no. AF6308) and Novus (Centennial, CO, USA) polyclonal biotinylated anti-collagen IV antibody (cat. no. NBP1-26550) followed by streptavidin-HRP. Collagen VI was quantified using a Santa Cruz HRP-conjugated anti-type VI collagen monoclonal antibody (cat. no. Sc-377143HRP). Tetramethylbenzidine (TMB) reagent was used for detection. Total insoluble (cytoskeletal) proteins were quantified using the sulforhodamine B method to standardize collagen signals to cell numbers [94]. Magnesium ascorbyl phosphate (MAP) was used as a positive control and sterile distilled water was the negative control. All colorimetric measurements were performed using the Molecular Devices (San Jose, CA, USA) microplate reader MAX190 and SoftMax3.1.2PRO software.

### 4.12. Effect of THDC (±AZ) on MMP Activity

The effects of THDC (±AZ) on the activity of MMP-1, MMP-2, MMP-3 and MMP-12 were evaluated. 0.1 g of sample was dissolved in 1 mL DMSO and diluted 100-fold with MMP buffer (50 mM Tris, pH 7.5, 150 mM NaCl, 2 mM CaCl_2_, 5μM ZnSO_4_, 0.01% Brij-35) to make stock solution. Serial dilutions of the sample (1-to-1) were then made to determine the IC_50_. Sample and 5 ng/well MMP-1 enzyme (Anaspec, Fremont, CA, USA, cat. no. AS-72004), MMP-2 enzyme (Anaspec, cat. no. AS-72005), or MMP-3 enzyme (Anaspec, cat. no. AS-72006) were incubated at 37 °C for 10 min. After 10 min, we added 0.2 μM/well 520 MMP FRET Substrate XIV (QXL^®^ 520-γ-Abu-P-Cha-Abu-Smc-HA-Dab(5-FAM)-AK-NH2 (Smc = S-Methyl-L-cysteine)) (Anaspec, cat. no. AS-60581). The fluorescence signal was monitored at an excitation of 485 nm and emission of 530 nm. To evaluate MMP-12 activity, sample was mixed with 0.2 mM/well AAAPVN (N-Succinyl-Ala-Ala-Ala-p-nitroanilide) substrate (Sigma, cat. no. S4760), 139 ng/well elastase (Alfa Aesar, Haverhill, MA, USA, cat. no. J61874) and readings were obtained following previously described methods [95].

### 4.13. Effect of THDC (±AZ) on Survival of H_2_O_2_-Stressed KCs

We evaluated effects of THDC (±AZ) on the viability of HaCaT KCs incubated with hydrogen peroxide. The project design was based on previously described methods [96]. Test materials were dissolved in DMSO at 20 mg/mL with further dilutions made using sterile distilled water. Samples were added to exponentially growing adult HaCaT keratinocytes (Addexbio, San Diego, CA, USA) cultured in a 96 well plate in DMEM and 10% FBS. Test materials were added 1 h before H_2_O_2_ (10 mM) and the incubation period in the presence of test substances was an additional 1 h. After this time, cells were rinsed and viability was determined by the neutral red uptake assay with a Molecular Devices microplate reader MAX190 and SoftMax3.1.2PRO software.

## Data Availability

Raw and processed microarray data will be submitted to the Gene Expression Omnibus (GEO) database upon acceptance of this manuscript. All other raw data are available from the corresponding author upon request.

## Supplementary Materials

The following are available online at https://www.mdpi.com/article/10.3390/ijms22168756/s1, Figure S1. Effects of THDC (±AZ) on collagen IV and VI proteins. Figure S2. Microarray fluorescent pseudoimages. Figure S3. Affymetrix quality control metrics. Figure S4. Differential expression analyses.

## Abbreviations

AA	L-Ascorbicacid
AAPH	2,2′-Azobis(2-amidino-propane)dihydrochloride
aHDF	adulthumanadultdermalfibroblasts
AZ	acetylzingeroneBP
CTL	control
DEG	differentiallyexpressedgene
GO	geneontology
HE	hydroethidine
HORAC	hydroxylradicalscavengingcapacity
HPLC	high-performanceliquidchromatography
IC50	half-maximalinhibitoryconcentration
KC	keratinocyte
KEGG	kyotoEncyclopediaofgenesandgenomes
MDA	malonaldehyde
MMP	matrixmetallopeptidase
nHDF	neonatalhumanadultdermalfibroblasts
NORAC	peroxynitritescavengingcapacity
NUSE	normalizedunscaledstandarderrors
ORAC	oxygenradicalabsorbancecapacity
RHE	reconstitutedhumanepidermis
RLE	relativelogexpression
SOAC	singletoxygenscavengingcapacity
SORAC	superoxideanionscavengingcapacity
THDC	tetrahexyldecylascorbate
UVA	ultravioletAlight
UVB	ultravioletBlight
