# Supplementary material for: Tetrahexyldecyl Ascorbate (THDC) Degrades Rapidly under Oxidative Stress but Can Be Stabilized by Acetyl Zingerone to Enhance Collagen Production and Antioxidant Effects
Source: Int J Mol Sci. 2021 Aug 15;22(16):8756. doi: 10.3390/ijms22168756 (PMC8395926; doi:10.3390/ijms22168756)
Supplement: Supplementary file 1 [file ijms-22-08756-s001.zip › Supplement.pdf]

Supplemental Figures

Tetrahexyldecyl ascorbate (THDC) degrades rapidly under oxidative stress but can be stabilized by acetyl zingerone to enhance collagen production and antioxidant effects

William R. Swindell[1§], Manpreet Randhawa[2], Geovani Quijas[3], Krzysztof Bojanowski[3], Ratan K. Chaudhuri[2]

[1]The Jewish Hospital, Department of Internal Medicine, Cincinnati, OH, 45236, USA.

[2]Sytheon Ltd., Boonton, NJ, USA.

[3]Sunny BioDiscovery Inc., Santa Paula, CA, USA.

[§]Corresponding Author.

Supplemental Figure Legends

Figure S1. Effects of THDC (± AZ) on collagen IV and VI proteins. (A-D) COL IV protein. (E-H) Col VI protein. COL IV and VI proteins were measured in neonatal and adult human dermal fibroblasts (nHDF, aHDF). Proteins were measured in cellular and extracellular (media) fractions with magnesium ascorbyl phosphate (MAP) used as a positive control (n = 6-16 per group). Colorimetric signals were first normalized to cell numbers (sulforhodamine B assay) and then normalized to the non-treated CTL group. Treatments not sharing the same letter differ significantly ($P < 0.05$, Fisher's least significant difference; *$P < 0.05$, compared to CTL group).

Figure S2. Microarray fluorescent pseudoimages. The Affymetrix oligonucleotide fluorescent intensity is shown for each of the 20 samples. Darker colors correspond to more intense signals. The size of each chip is 1.28 cm x 1.28 cm (scale bar = 4 mm).

Figure S3. Affymetrix quality control metrics. (A) A260/A280 ratios. Ratio values of approximately 2.0 are consistent with high purity RNA. (B) Probe-level model residuals. Boxes span the middle 50% of residual values for each array (i.e., 25th to 75th percentiles), and whiskers span the 10th to 90th percentiles. (C, D) Normalized Unscaled Standard Errors (NUSE) median and IQR. Median NUSE values greater than one or high NUSE IQR values suggest poor quality arrays. (E, F) Relative log expression (RLE) median and IQR. Median RLE values not near zero or large RLE IQR values suggest poor quality arrays. (G) Percentage of outlier genes. The percentage of outlier genes on each array was detected using the outForest algorithm. (H) Cluster analysis. The 20 samples were clustered using the average linkage method and Euclidean

distance between normalized expression vectors. (I) Principal component (PC) plots. The 20 samples were plotted with respect to the first two PC axes.

Figure S4. Differential expression analyses. (A, B) P-value distributions. The p-value distribution is shown for each comparison. The total number of DEGs identified (increased and decreased) is indicated in the top margin. (C, D) Volcano plots. The -log10-transformed p-value is plotted with respect to fold-change estimate. (E, F) MA plots. Fold-change is plotted with respect to average expression. The green line represents the loess regression fit. (G, H) Average expression in each treatment. Each gene was normalized such that average expression was equal to 1 among all 20 samples. Contour lines generated from multivariate kernel density estimation are shown in yellow. In (C) - (H), the number of DEGs identified is indicated in the top margin (red = increased; blue = decreased).

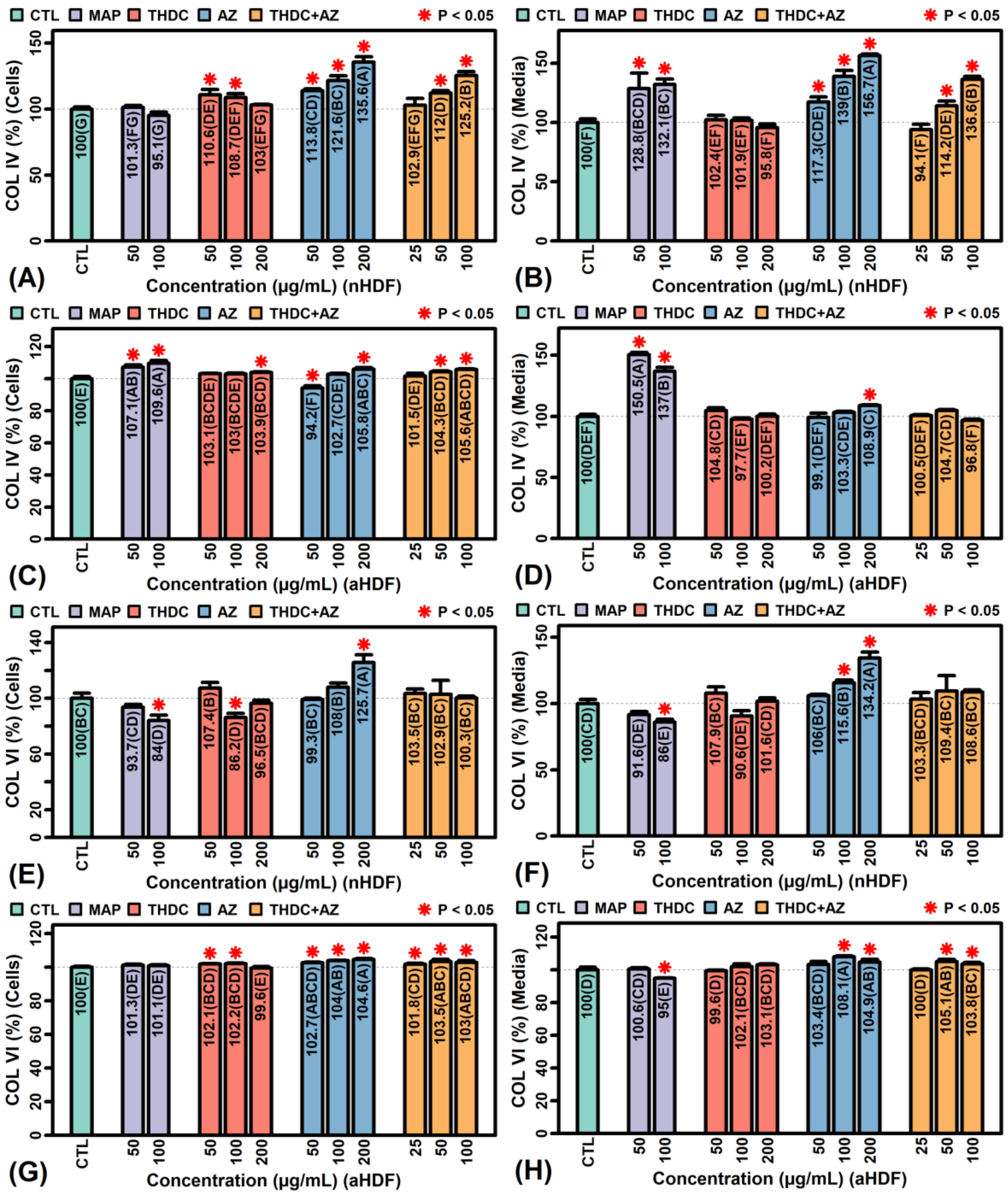

Figure S1. Effects of THDC (± AZ) on collagen IV and VI proteins.

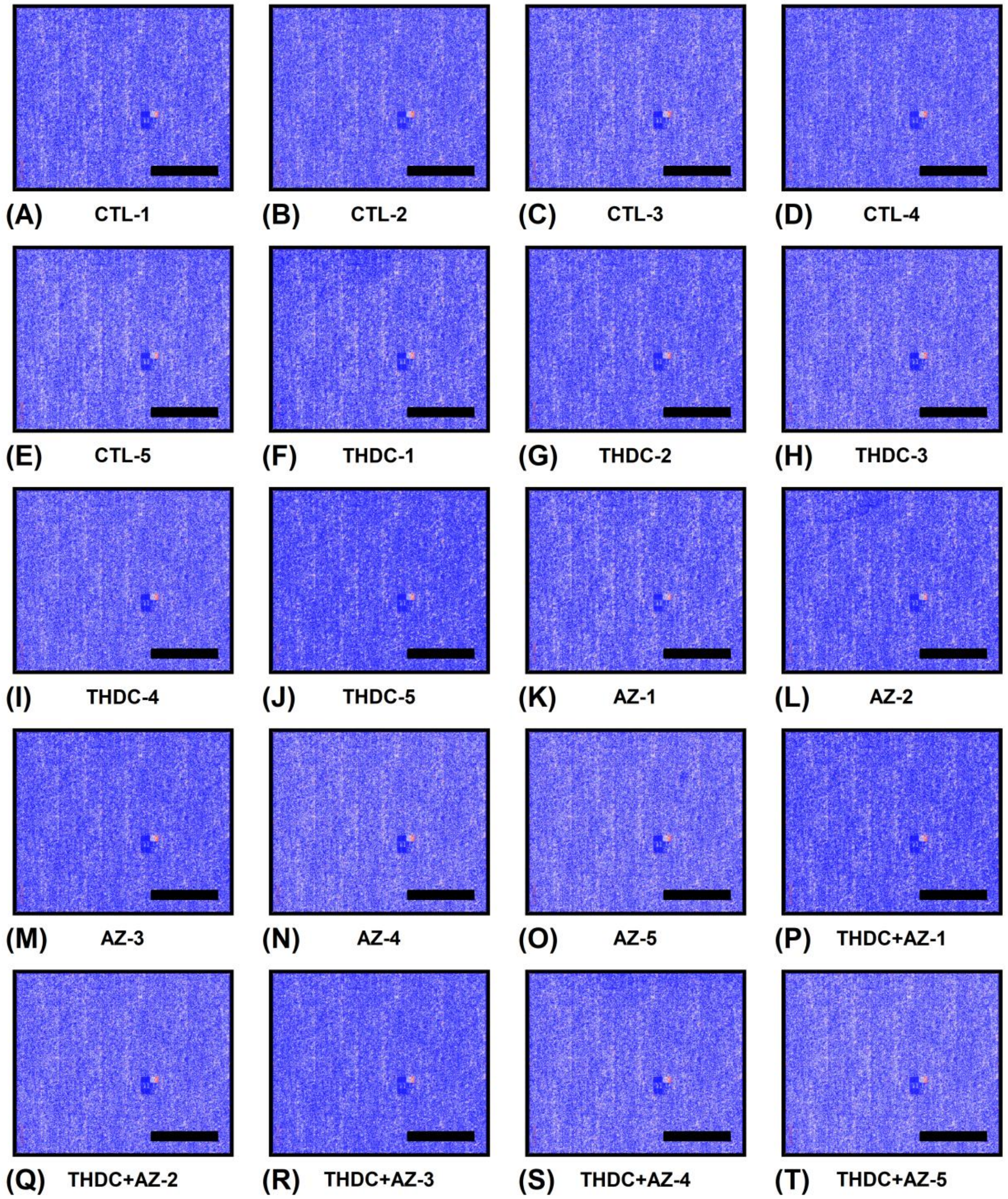

Figure S2. Microarray fluorescent pseudoimages.

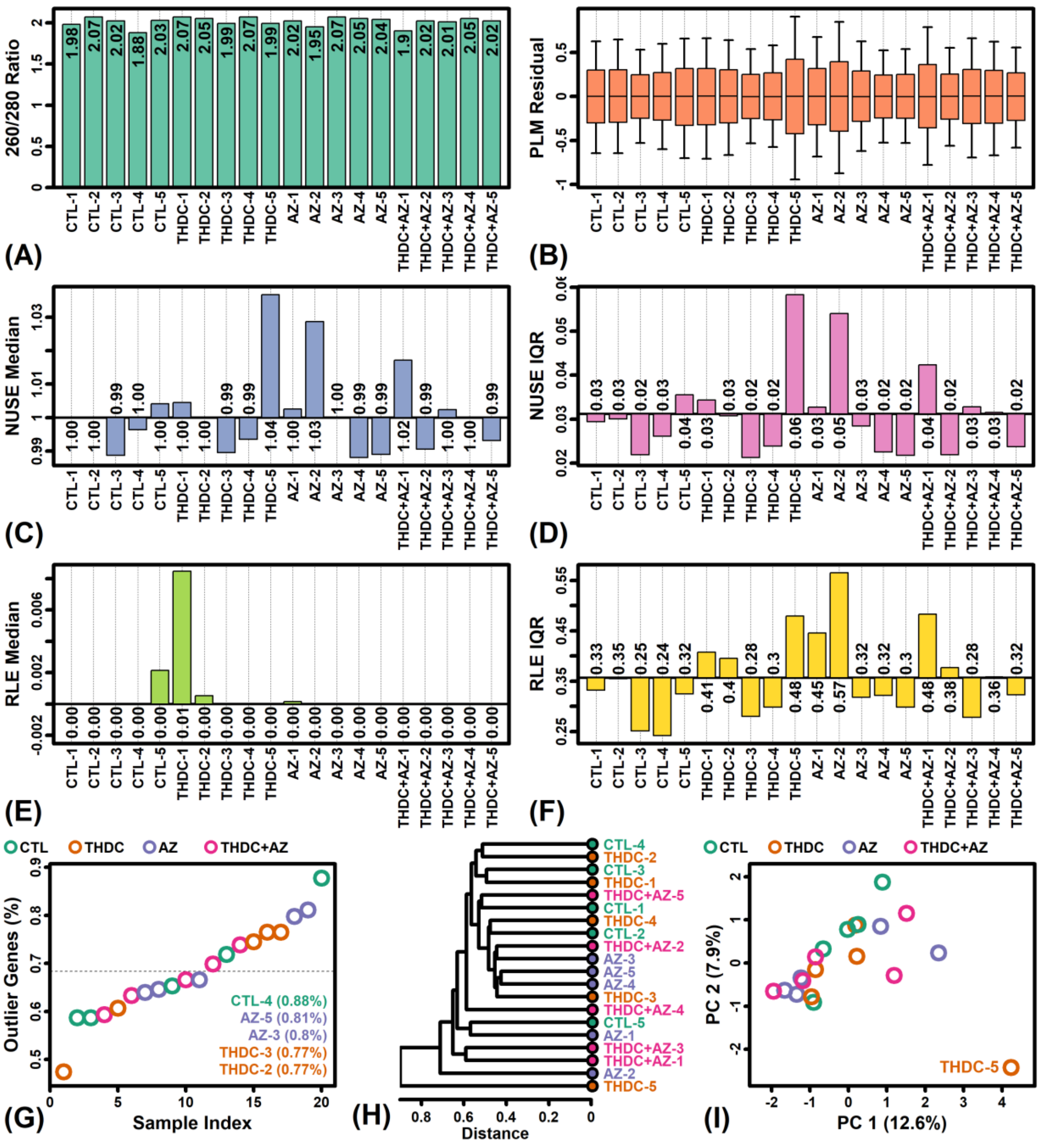

Figure S3. Affymetrix quality control metrics.

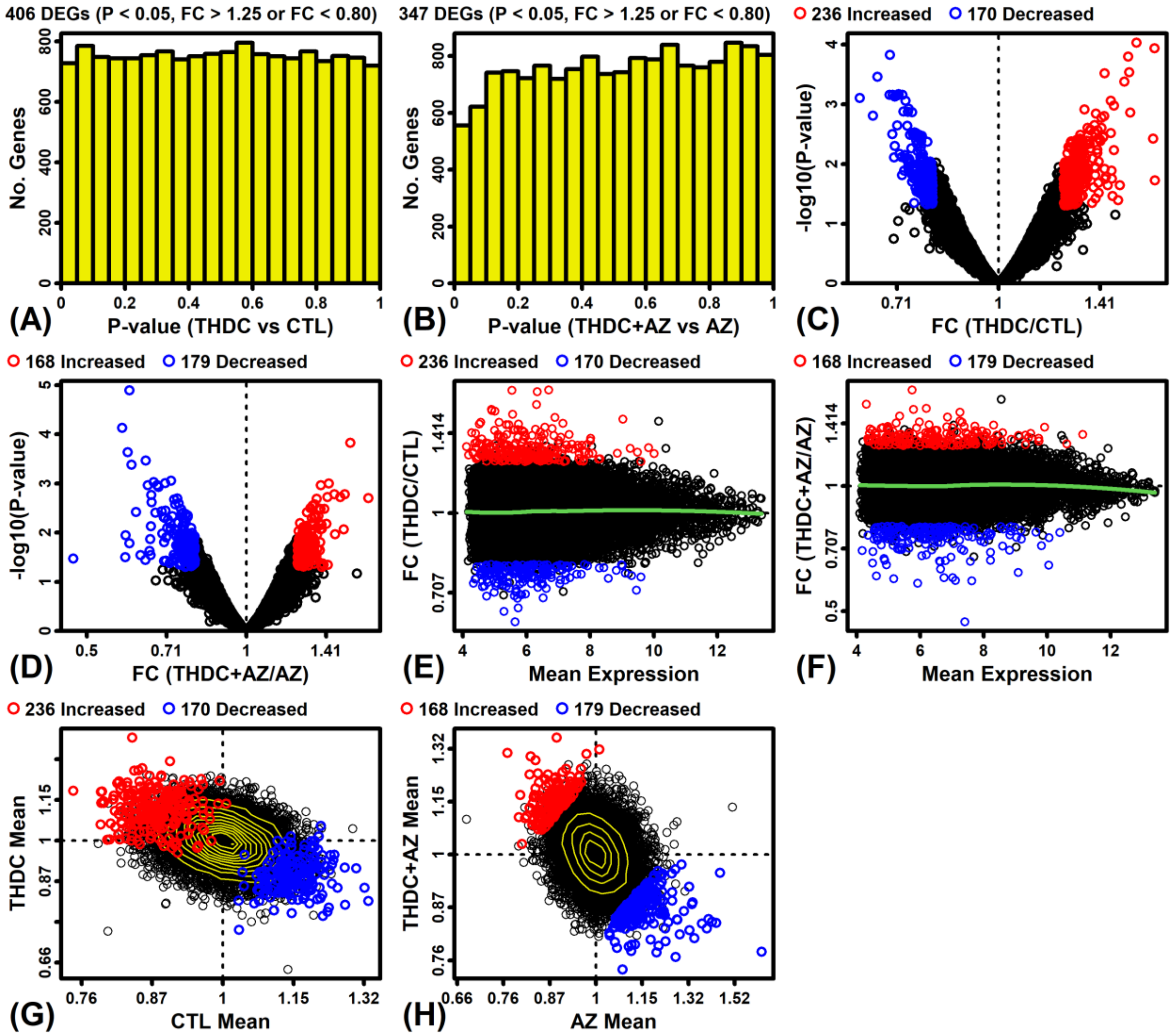

Figure S4. Differential expression analyses.
